# Bibliographical Notices

**Published:** 1849-04

**Authors:** 


### BIBLIOGRAPHICAL NOTICES.

*Miscellaneous Publications.*—Under this head we shall proceed to enumerate various publications with which we have been favored, indulging comments upon some of them, and, with respect to others, being obliged to express, in this way, with more brevity than would suit our inclinations, acknowledgements of the courtesy of their respective authors, or publishers.

*Braithwaite's Retrospect.* Part the eighteenth. New York. Daniel Adee. 107 Fulton st. 1849.

*Raking's Abstract.* No. 8. Philadelphia. Lindsay & Blackiston. 1849,

These half-yearly publications are too well and generally known to require remark, save that they do not appear to diminish in value.

*Report of the Eastern Asylum in the City of Williamsburg, Virginia,* 1848. Richmond. 1849.

John M. Galt, M. D., is Physician and Superintendent of this Asylum for the insane. The Institution is to be enlarged; the sum of twenty-five thousand dollars having been appropriated by the State for that purpose. The Report of Dr. Galt contains a description of the plan and architectural style of the new buildings to be erected. The Institution is in a flourishing condition.

*Report of the Pennsylvania Hospital for the Insane, for the year* 1848. By Thomas S. Kirkbride, M. D. Physician to the Institution. Published by order of the Board of Managers. Philadelphia. 1849.

This Institution will accomodate about two hundred patients. Dr. Kirkbride is of opinion that this number is as many as can be visited daily by one medical superintendent, and that a larger number should not be collected in any one building. In this we think he is right.

The following suggestion is to us novel:—

"*Detached Cottages for Patients.*—The additional experience acquired since the date of my last report, goes fully to confirm the views expressed a year ago, in reference to the value of a few detached cottages in connection with any hospital for the insane that receives all classes of patients. Where persons have ample means, there can be no reason why, when insane, they should not have around them every comfort, or even harmless luxury, which wealth can procure, or to which they have been accustomed. There can be no reason why, if they fancy it, they should not be select in their associates; why they should not have educated and refined companions constantly with them, nor why they should not have apartments entirely private, in which they can receive their friends and relatives whenever it is deemed advisable."

*Sixteenth Annual Report of the Trustees of the State Lunatic Hospital at Worcester, Mass.* Dec. 1848.

Geo. Chandler, M. D., is Physician and Medical Superintendent of this Institution.

The year commenced with three hundred and ninety-four patients. The smallest number, at any time, was three hundred and eighty-eight. The average number for the year was four hundred and four. This number was too great for the accommodations, irrespective of the difficulties of proper medical supervision of, individually, so large a number. Dr. Chandler is opposed to farther enlargement of the hospital, and advocates the erection of another hospital in the western part of the State. He also suggests the propriety of devoting a hospital exclusively to females.

*Report of the Board of Visitors of the Boston Lunatic Asylum in the Matter of the Superintendent of that Institution.* 1849.

The circumstances giving occasion for this report, were as follows: Dr. Stedman, Physician to the Hospital, it appears, after examination of the case of the wife of Dr. Kraitsir, of Boston, regarded the case as one of insanity, and gave a certificate of his opinion to that effect. For reasons which are not stated in the report, and of which we have heard nothing, the Common Council of the city instructed the Board of Visitors to cause an immediate investigation of the circumstances which induced Dr. S. to give this certificate.

In the communication of Dr. S. to the Board, he takes an independent, manly position with respect to the right of the City Council to make a mandatory requisition upon him for explanation of professional conduct in his private capacity, having no connection with his public duties. He assumes that if the requisition is made under suspicion of his having been guilty of bribery, corruption, or conspiracy, the judicial courts, or the grand jury, are the tribunals to which he should be called upon to answer; and if the inquiry is made irrespective of any such suspicion, it is a matter of his own, concerning which he is under no obligations to answer. Nevertheless, having been publicly called upon for the statement, and in consideration that his silence might furnish occasion for the calling in question, by the public, of his humanity and integrity, he accedes to the mandate. The explanation appears to have been satisfactory. The complaint, as in many other instances of the same description, seems to have originated in gossip, or malice, which, when explained, leaves the authors in a ridiculous predicament.

Dr. S. is entitled to credit for taking a stand, due alike to himself, and to the character of the profession. The freedom from servility to those upon whom his tenure of office depends, which he displays, is good evidence that he is well deserving the honorable and responsible situation which he occupies.

*House of Representatives. Dr. Edwards, from the Select Committee, to whom was referred the Memorial of W. T. G. Morton, asking Compensation from Congress for the Discovery of the Anæsthetic or Pain Subduing Property of Sulphuric Ether. Report.*

Before this committee, the proofs of originality, or rather priority, in the application of the vapor of ether to anæsthetical purposes, were submitted by Drs. Jackson and Morton, (each of whom appeared personally before the committee, and also Mr. H. L. Lord, on behalf of Dr. Jackson,) and the committee decided in favor of the claims of the latter, recommending an appropriation, but not fixing upon any sum. The committee say that they are satisfied "that Dr. Morton is entitled to the merit of the discovery. *The great thought was of producing insensibility to pain, and the discovery consisted in that thought, and in verifying it practically by experiment.* (This passage is italicised in the report.) For this, the world is indebted to Dr. Morton," etc.

It does not appear that the committee investigated the claims of the late Dr. Wells, of Hartford, Conn., to the discovery. He is alluded to, in the Report, as having made an unsuccessful experiment with the nitrous oxide, in Boston, anterior to the experiments of Morton. The fact of the experiment, albeit unsuccessful, shows that he entertained the "great thought" referred to in the foregoing paragraph. But is it not abundantly established by reliable witnesses, that he had made, previous to this unsuccessful experiment at Boston, successful experiments with the nitrous oxide, and on one occasion, even with the ether; and that surgical operations had been performed upon patients rendered insensible by him? If so, why is he not to be regarded as the discoverer? It seems to us a plain case, although we have no wish to detract from any merit which may attach to Drs. Jackson or Morton, either or both, for their subsequent, and, so far as relates to bringing the subject before the profession and public, more successful labors. With respect to the rival efforts of the two latter gentlemen, and their respective friends, the prospect of their agreement, or the triumph of either party, seems more remote than the discovery of the source of the Niger. We can see no better way than to decide from their own affidavits, when they applied for a patent, in which, (if we remember rightly) they make oath that the discovery was jointly made.

*Jarvis on Dislocation of the Shoulder.*—A pamphlet of 32 pages, setting forth the efficacy of the *adjuster*, invented and patented by Dr Jarvis, in reducing luxations of the shoulder. Compiled by GEORGE KELLOG, A. M. of Derby, New Haven Co. Conn.

*A Discourse on the Influence of Diseases on the Intellectual and Moral Powers.* By JOSEPH MATHER SMITH, M. D. Professor of Theory and Practice of Physic and Clinical Medicine, College of Physicians and Surgeons, N. Y.

An introductory lecture on a philosophical subject, philosophically treated, and well deserving attentive perusal by lay readers, as well as physicians.

*An Introductory Lecture, delivered before the Students of Memphis Medical College.* By GEORGE R. GRANT, M. D. Professor of the Theory and Practice of Medicine. Published by the class.

A sensible discourse, pointing the student to the bright examples of distinction and usefulness in the medical profession, which offer themselves for imitation.

*An Essay on Strictures of the Urethra, with an account of the mode of curing strictures by cutting.* By JAMES BRYAN, M. D. Philadelphia.

A paper of 21 pages. The subject is fully expressed in the title. Of the merits of the suggestions offered we do not presume to judge.

*On Etherization: a Paper read before the Medical Society of Louisville.* By LUNSFORD P. YANDELL, M. D. Prof of Chemistry and Pharmacy in the University of Louisville. Published by the Society. pp. 36.

An excellent summary of reliable facts gathered from various sources, respecting the safety of etherization, and its various applications to surgery and disease. It must have cost the author considerable labor, and is very opportunely presented to the profession.

*Annual Address delivered before the New York State Medical Society, and Members of the Legislature, at the Capitol.* By ALEXANDER H. STEVENS, M. D. President of the American Medical Association, of the College of Physicians and Surgeons at New York City, and of the State Med. Society, etc.

This address is entitled "A Plea in Behalf of Medical Education." The appreciation of it by our legislators at Albany, before whom, together with the members of the State Society present at the annual meeting, it was delivered, is evinced by a resolution of the senate to print it for distribution among themselves. The Medical Society also ordered an issue in a separate form, as well as in connection with "their Transactions." These are high compliments, but not greater than the production deserves. It is a high toned, well written address, and, we can well conceive, was impressively delivered. The author shows what medical men have done, and are doing in the cause of humanity; and thence deduces the claims of medical education upon the Public. He advocates a system of free education in medicine, urging the Legislature to endow medical colleges, appropriate salaries from the state treasury, and open the doors without charge.

*Anniversary Discourse before the New York Academy of Medicine.* By JAMES R. MANLEY, M. D. Published by order of the Academy.

Dr. Manley appears to be of that class who are under painful apprehensions that the medical profession of this country, already degraded by the ignorance and incompetency of its members, is constantly deteriorating, chiefly in consequence of the malversations of incorporated medical schools. The Doctor's remedy, we suppose, would be to divest diplomas granted by medical institutions of any virtue, as a license to practice, and entrust the bestowal of testimonials of qualifications to a board of examiners, composed of practitioners not engaged in teaching. We do not purpose to discuss the propriety of such a change. It is perhaps sufficient to say that such boards do already exist, in the State Censors for the different districts of this state. Is it not a matter of surprise that so few candidates appear before these boards, if the plan of separating licensing and teaching possesses the recommendations claimed for it? At all events, the small number licensed by state and county censors, and the proportion of those licensed who subsequently resort to the means necessary to secure diplomas, sufficiently show the general opinion of the profession, as well as of the public, on this subject.

*Lecture on Obstetrics and the Diseases of Women and Children.*—By GUNNING S. BEDFORD, M. D. Prof. of Obstetrics in the University of New York.

In this discourse the author sustains the reputation acquired by previous productions of a similar character.

*An Address, Delivered at the Opening of the Rock Island Medical School.* By M. L. KNAPP, M. D. President, and Prof. of Materia Medica and Therapeutics.

This production invites criticism, but we decline the invitation.

*An Account of some of the Most Important Diseases Peculiar to Women.* By ROBERT GOOCH, M. D. With illustrations. Second edition. Philadelphia. Ed. Barrington and Geo. D. Haswell. 1848. 8 vo. pp. 322.

This, although not a new book, will amply repay perusal by those not already familiar with its contents. Dr. Gooch was an original thinker, and an excellent practical observer. If we mistake not, to him is due the credit of first directing attention to that morbid condition in children in which congestion of brain is simulated—the hydrencephaloid disease, of Marshall Hall. A chapter of this volume is devoted to that subject. His observations on irritable uterus, on the first publication of the work, excited much attention. We suppose the affection described by him is now appropriately to be classed among the varied phenomena of what is ordinarily called spinal irritation. The remaining subjects treated of in the work are, peritoneal (puerperal) fevers; disorders of the mind in lying-in women; thoughts on insanity; mode of discriminating pregnancy from the diseases which resemble it; polypus of the uterus, and excrescencies liable to be mistaken therefor.

We would especially commend to the attention of the medical student and young practitioner, the advice which the author gives in the preface, touching the means of acquiring useful, practical knowledge in medical science.

*A System of Clinical Medicine.* By ROBERT JAMES GRAVES, M. D., M. R. J. A., etc. With notes, and a series of lectures, by W. W. GERHARD, M D., etc. Third American Edition. Philadelphia: Ed. Barrington & Geo. D. Haswell. 1848. 8 vo. pp. 751.

"The demand for a new edition" (as the publishers remark in the preface) "is the best proof of the high character these lectures have gained in the United States, and of the very extended reputation they have justly attained." The present edition is printed from a new one, considerably enlarged, published by Dr. Graves, in Europe, since the preceding American Edition was issued. Some detached papers, however, included in the last European Edition, are omitted, and will, probably, be issued by the publishers in a separate form.

The work is eminently practical in its character. It commends itself, as containing the clinical views of two distinguished teachers, both ranking among the ablest pathologists, and the most judicious practitioners, the profession affords.

*An Illustrated System of Human Anatomy, Special, General and Microscopic.* By SAMUEL GEORGE MORTON, M. D., Penn. and Edinburg. Member of the Medical Societies of Philadelphia, New York, Boston, Edinburg and Stockholm; author of Crania Americana, Crania Ægyptiaca, etc. With three hundred and ninety-one engravings on wood. "Occulis subjecta fidelibus." Philadelphia: Grigg, Elliot & Co., No. 14 North Fourth Street. 1849. pp. 642.

We welcome this volume with pleasure, and, as an American work, with pride. Its beautiful typography, and well executed illustrations, are highly creditable to the enterprising publishers, and, in so far as we have been able to examine it, it seems to us fully commensurate with what would be expected from the pen of the distinguished author. We are assured by those who have bestowed upon it more critical attention than our engagements have as yet permitted us to do, that, as a text book of anatomy, it is all that could be desired. We had been promised an extended review by an able correspondent, but, much to our disappointment, it has not been received. If the little we have said, should, perchance, determine any of our readers to purchase the work, we doubt not they will feel obliged to us.

